# Changes in weight and body composition across five years at university: A prospective observational study

**DOI:** 10.1371/journal.pone.0225187

**Published:** 2019-11-13

**Authors:** Tom Deliens, Benedicte Deforche, Laurent Chapelle, Peter Clarys

**Affiliations:** 1 Department of Movement and Sport Sciences, Vrije Universiteit Brussel, Pleinlaan, Brussels, Belgium; 2 Department of Public Health and Primary Care, Ghent University, C. Heymanslaan, Ghent, Belgium; McMaster University, CANADA

## Abstract

**Objectives:**

The transition from high school to college or university has been shown to be a critical period for weight and fat gain. To date, no European data on weight and fat gain over the full trajectory of college or university are available. Therefore, the purpose of the present study was to investigate weight and fat gain among Belgian university students from freshman through senior year of university.

**Methods:**

In this prospective observational study, a total of 340 university students were measured six times, i.e. at baseline (start of the first academic year) and at the start of the second semester of the first, second, third, fourth and fifth academic year. Objective measurements included body weight, height, body mass index (BMI), fat%, fat mass, fat free mass and waist circumference. Multilevel modelling was used to assess anthropometric changes over time according to sex.

**Results:**

From freshman through senior year of university, individual weight changes ranged from -5.2 to +15.7kg, and respectively 77.4% and 69.3% of students showed increases in BMI and fat%. Stronger increases in weight and BMI were found for males (5.7kg; 1.6kg/m^2^; both *p*<0.001) than females (2.2kg; 0.8kg/m^2^; both *p*<0.001) over the 4.5 year measuring period. Similarly, waist circumference increased more in males (4.7cm; *p*<0.001) than in females (1.0cm; *p*<0.001). In contrast, females (2.5%; *p*<0.001) showed a higher increase in fat% than males (1.0%; *p* = 0.019). Across sexes, the highest weight and fat gains were found during the first semester and during the final year of university.

**Conclusions:**

Belgian university students gain a significant amount of body weight and body fat from freshman through senior year of university. Similar to the first semester, more pronounced increases in weight and fat were observed during the final year of university. Interventions aiming at preventing weight gain at university should not only focus on freshmen but also on senior students.

## Introduction

The transition from high school to college or university has been shown to be a critical period for weight and fat gain [1, 2]. The most recent meta-analysis on freshman weight gain calculated a mean weight increase of 1.36 kg over an average of five months [2]. It was also concluded that students who gained weight, gained it at rates much faster than in the general population [2]. Another meta-analysis of Fedewa and colleagues [1] showed that this weight gain is generally accompanied by an increase in fat%. To date, research mainly focused on freshman weight gain, while, to the best of our knowledge, only one US study investigated weight combined with fat gain from freshman through senior year of college. This study reported a 4-year weight gain of 3.0 kg, while weight gains were greater in males (5.9 kg) than in females (1.7 kg) and were accompanied by increases in fat% [3].

European literature assessing weight and fat gain trends beyond the first year of college or university is scarce. In previous research [4] we concluded that male Belgian university students gained 2.7 kg of weight and showed an increased waist circumference (WC) of 1.8 cm across 1.5 years at university. In contrast, no changes were observed in female students over the same measuring period. We further concluded that the largest weight and fat gains occurred during the first semester at university. To date, no European data on weight and fat gain over the full trajectory of college or university are available. Due to socio-cultural and environmental factors (e.g. fast food and all-you-can-eat dining culture, supersized foods and drinks, etc.) the above US findings cannot be generalized to the European university student population. It is, however, important to know how this student weight gain evolves throughout the years at university in order to enable weight gain prevention strategies to focus on the right target group and period of risk. Therefore, the purpose of the present study was to expand on previous research [4] and to provide follow-up results on weight and fat gain among Belgian university students across five years at university.

## Materials and methods

### Design and participants

In this longitudinal study, 348 Belgian (Flemish) first year student volunteers were recruited via e-mail and face-to-face on the university campus (Vrije Universiteit Brussel, Brussels, Belgium). Two-hundred and seven of them were recruited at the start of the academic year in 2011 (September/October), while 141 students were recruited at the start of the following academic year, that is September/October 2012. Only students who had just finished their last year of high school and were in their first year of university (so called generation students) were eligible to participate. Of the 348 student volunteers, 340 were identified as generation students and thus eligible to participate. The representativeness of the sample (n = 340) could be determined by comparing the sample characteristics with self-reported data of all first year university students (n = 926) which were collected by the university’s registration office during students’ enrolment. Our sample with mean age of 18.0 ± 0.6 years, 59.1% females and mean body mass index (BMI) of 21.4 ± 2.7 kg/m^2^ was representative compared to all freshmen in sex (56.6% females; *chi^2^* = 0.6, *p* = 0.431) and BMI (21.4 ± 2.8 kg/m^2^; *t* = 0.2, *p* = 0.851). Mean age was slightly higher in the total group of freshmen (18.5 ± 1.1 years; *t* = 9.8, *p*<0.001).

### Procedure

Baseline measurements (T0) were conducted at the start (October/November) of the first semester of students’ first academic year at university. Participating students were measured again at the start of the second semester (February/March) of the first (T1), second (T2), third (T3), fourth (T4) and fifth (T5) academic year. So, the study ran from freshman through senior year and comprised a total measuring period of 4.5 years. For each test occasion (n = 6), participants were contacted via telephone and/or e-mail to make an appointment at a time that was convenient for them and fitted their class schedule. Participants’ timing of measurement at baseline was taken into account as much as possible during the follow-up measurements. The researchers made up to ten attempts on different moments during the day to contact the students before they were considered to have dropped out. To minimize possible effects owing to sudden short-term lifestyle changes after holiday periods, measurements were conducted after two weeks of academic activities. After each measurement, students received two incentives, namely a lunch voucher which they could spend in the on-campus restaurant, as well as a personalized report of their own anthropometrics. All participants were informed about the nature of the study and written consent was obtained before the start of the study. The study protocol was approved (B.U.N. 143201111941) by the Medical Ethics Committee of the university hospital (Vrije Universiteit Brussel, Brussels, Belgium).

### Measurements

The measurements were performed by trained researchers under the supervision of the principal investigator. Students were measured with bare feet, wearing shorts and a t-shirt. They were asked to void their bladder before starting the measurements, as well as to remove all jewellery. Weight (TANITA BC-418 Body Composition Analyzer, Tanita Corp., Tokyo, Japan) and height (wall-fixed stadiometer) were measured to calculate BMI. Body composition, including fat%, fat mass (FM) and fat free mass (FFM), was estimated using TANITA BC-418 Body Composition Analyzer, which is a validated eight-electrode device for hand-to-foot bioelectrical impedance analysis operating with a 50-kHz current [5]. Participants were instructed to step on the electrodes with bare feet and to grab both hand grips. WC was measured with Cescorf Anthrotape on the narrowest part of the waist to analyse body fat distribution [6]. All measurements, except those using TANITA, were conducted twice and an additional third time when tolerance limits were exceeded. Subsequently, mean values were calculated from the two nearest values.

### Statistical analyses

SPSS Statistics 24 and R (RStudio version 1.1.383) were used for data analyses. Independent samples *t*-tests and *chi^2^*-tests were performed in SPSS to analyse representativeness of the sample and drop-out. Multilevel modelling was performed in R to assess anthropometric changes across five years at university. The model included two levels, i.e. the individual level and the study discipline level, which were included as random factors. Time was included as a fixed factor. As previous research showed that anthropometric changes among Belgian students were sex-dependent [4, 7, 8], both sex and the interaction term time x sex were also included as fixed factors. The maximum-likelihood (ML) method was used, as ML produces more accurate estimates of fixed regression parameters (which are the main interest of this study) compared to the ‘default’ restricted maximum-likelihood (REML) method [9, 10]. *P*-values <0.05 were considered as statistically significant, whereas *p*-values <0.1 were considered as trends towards significance.

## Results

### Drop-out analysis

**Fig 1:**
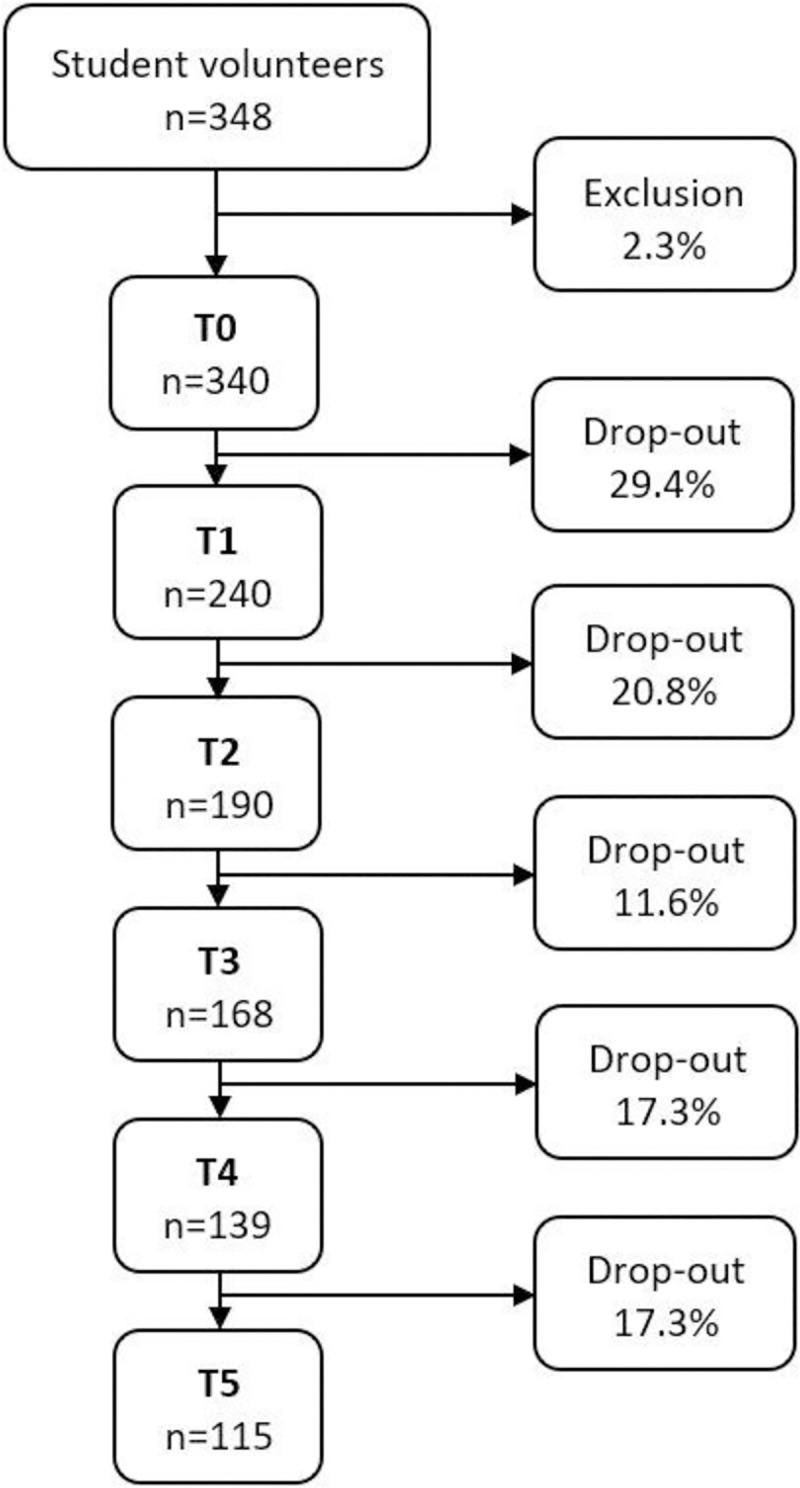
Flow chart with drop-out rates across the measurements.

Of the initial sample (n = 340; T0), 115 students (60.9% females) were measured during senior year of university (T5; see Fig 1). Reasons for drop-out were not reachable, quit university, no longer interested in participating, or graduated (as some Master studies have a 4-year trajectory). Drop-out analysis was conducted to ensure representativeness of the retention sample. At baseline, both retention and drop-out group were similar in sex (60.9% vs. 58.2% females; *chi^2^* = 0.221, *p* = 0.639), age (17.9 ± 0.7 vs. 18.0 ± 0.5 years; *t* = 1.2, *p* = 0.231) and BMI (21.1 ± 2.2 vs. 21.5 ± 2.8 kg/m^2^; *t* = 1.3, *p* = 0.190). It should be mentioned that, regardless of missing values caused by drop-out, the multilevel modelling technique that we used in the present study automatically includes all students (n = 340) at every time point and therefore controls for drop-outs.

### Anthropometric changes across five years at university

Table 1 shows anthropometric mean estimates from freshman through senior year of university. Significant changes between each follow-up time point (T1-5) and baseline (T0) respectively, are indicated by superscript a, while changes between subsequent time points are indicated by superscript b. Although results across year 1 and 2 (T0-T2) have been presented elsewhere (Deliens et al., 2015), we opted to display all time point results in the present study, as we aimed to depict the whole 5-year trajectory. It should also be mentioned that, in the current study, we performed a different type of statistical analysis providing mean estimates which may slightly differ from the means presented in our previous work.

**Table 1:** Anthropometric mean estimates (SE) from freshman through senior year of university.

Measurements	Start 1^st^ semester of the 1^st^ year (T0)	Start 2^nd^ semester of the 1^st^ year (T1)	Start 2^nd^ semester of the 2^nd^ year (T2)	Start 2^nd^ semester of the 3^rd^ year (T3)	Start 2^nd^ semester of the 4^th^ year (T4)	Start 2^nd^ semester of the 5^th^ year (T5)
Weight (kg)						
Males (n = 139)	69.9 (0.9)	71.2 (0.9)^a^^,^^b^	72.6 (0.9)^a^^,^^b^	73.5 (1.0)^a^^,^^b^	74.2 (1.0)^a^^,^^	75.6 (1.0)^a^^,^^b^
Females (n = 201)	58.5 (0.8)	59.1 (0.9)^a^^,^^b^	58.9 (0.9)	59.2 (0.9)^a^	59.9 (0.9)^a^^,^^b^	60.7 (0.9)^a^^,^^b^
BMI (kg/m^2^)						
Males (n = 139)	21.6 (0.3)	22.0 (0.3)^a^^,^^b^	22.4 (0.3)^a^^,^^b^	22.6 (0.3)^a^^,^^b^	22.8 (0.3)^a^	23.2 (0.3)^a^^,^^b^
Females (n = 201)	21.1 (0.2)	21.3 (0.2)^#^^,^^	21.2 (0.2)	21.3 (0.2)^#^	21.6 (0.2)^a^^,^^b^	21.9 (0.2)^a^^,^^b^
Fat%						
Males (n = 139)	14.9 (0.6)	15.5 (0.6)^a^^,^^b^	15.0 (0.6)	13.9 (0.6)^a^^,^^b^	14.4 (0.6)	15.9 (0.7)^a^^,^^b^
Females (n = 200)	24.8 (0.5)	25.5 (0.5)^a^^,^^b^	25.4 (0.5)^a^	25.6 (0.5)^a^	26.5 (0.6)^a^^,^^b^	27.3 (0.6)^a^^,^^b^
FM (kg)						
Males (n = 139)	10.6 (0.5)	11.4 (0.6)^a^^,^^b^	11.2 (0.6)^#^	10.6 (0.6)^	11.1 (0.6)	12.5 (0.6)^a^^,^^b^
Females (n = 200)	14.9 (0.5)	15.5 (0.5)^a^^,^^b^	15.4 (0.5)^a^	15.6 (0.5)^a^	16.3 (0.5)^a^^,^^b^	17.1 (0.5)^a^^,^^b^
FFM (kg)						
Males (n = 139)	59.2 (0.5)	59.8 (0.5)^a^^,^^b^	61.4 (0.5)^a^^,^^b^	62.8 (0.6)^a^^,^^b^	63.0 (0.6)^a^	63.1 (0.6)^a^
Females (n = 200)	43.6 (0.5)	43.5 (0.5)	43.4 (0.5)	43.6 (0.5)	43.5 (0.5)	43.7 (0.5)
WC (cm)						
Males (n = 139)	75.7 (0.6)	76.9 (0.6)^a^^,^^b^	77.6 (0.6)^a^^,^^b^	78.4 (0.6)^a^^,^^b^	78.8 (0.6)^a^	80.4 (0.7)^a^^,^^b^
Females (n = 201)	68.1 (0.5)	68.1 (0.6)	68.0 (0.6)	68.1 (0.6)	68.5 (0.6)	69.1 (0.6)^a^^,^^

^a^ significantly different from baseline measurement (p<0.05)

# trend towards significant difference with baseline measurement (p<0.1)

^b^ significantly different from previous measurement (p<0.05)

^ trend towards significant difference with previous measurement (p<0.1)

BMI = body mass index; FM = fat mass; FFM = fat free mass; WC = waist circumference; SE = Standard Error

Across five years at university, individual weight changes ranged from -5.2 to +15.7 kg, and respectively 77.4% and 69.3% of students showed increases in BMI and fat%. Fifty-three percent of participants gained ≥5% of their initial body weight, which can be considered as clinically relevant [11]. From baseline (T0) to year 5 (T5) significant time x sex interactions were found for weight (*p*<0.001), BMI (*p*<0.001), fat% (*p* = 0.009), FFM (*p*<0.001) and WC (*p*<0.001). A stronger increase in weight was found for males (5.7 kg; *p*<0.001) than females (2.2 kg; *p*<0.001) from freshman through senior year of university. Similarly, higher gains in BMI (1.6 vs. 0.8 kg/m^2^; both *p*<0.001), FFM (3.9 vs. 0.1 kg; *p*<0.001 vs. *p* = 0.882) and WC (4.7 vs. 1.0 cm; both *p*<0.001) were found for males compared to females. In contrast, females (2.5%; *p*<0.001) showed a higher increase in fat% than males (1.0%; *p* = 0.019) across the 4.5-year measuring period. No time x sex interaction was found for FM (*p* = 0.508), meaning there was no significant difference between the magnitude of FM increase in males (1.9 kg; *p*<0.001) and females (2.2 kg; *p*<0.001).

Significant increases in weight (1.3 kg; *p*<0.001), BMI (0.4 kg/m^2^; *p* = 0.001), fat% (0.6%; *p* = 0.046), FM (0.8 kg; *p* = 0.007), FFM (0.6 kg; *p* = 0.012) and WC (1.2 cm; *p*<0.001) were observed among male students during the first semester at university (T0-T1). For females, a first semester increase was found for weight (0.6 kg; *p* = 0.031), fat% (0.7%; *p* = 0.009) and FM (0.6 kg; *p* = 0.008), while a trend towards a significant increase was observed for BMI (0.2 kg/m^2^; *p* = 0.076). No changes were found for FFM (*p* = 0.615) and WC (*p* = 0.853). Only in male students significant increases were found between year 1 and 2 (T1-T2), and year 2 and 3 (T2-T3) for weight (1.4 kg; *p*<0.001 and 0.9 kg; *p* = 0.028), BMI (0.4 kg/m^2^; *p* = 0.006 and 0.2 kg/m^2^; *p* = 0.036), FFM (1.6 kg; *p*<0.001 and 1.4 kg; *p*<0.001) and WC (0.7 cm; *p* = 0.037 and 0.8 cm; *p* = 0.023). Also, a significant decrease in fat% (-1.1%; *p* = 0.008) was found between year 2 and 3 (T2-T3). In females on the other hand, significant increases were found between year 3 and 4 (T3-T4) for weight (0.7 kg; *p* = 0.047), BMI (0.3 kg/m^2^; *p* = 0.029), fat% (0.9%; *p* = 0.023) and FM (0.7 kg; *p* = 0.016). Almost similar to the first semester results, consistent increases were found across sexes between year 4 and 5 (T4-T5). Increases were observed in weight males: 1.4 kg; *p* = 0.002; females: 0.8 kg; *p* = 0.026), BMI (males: 0.4 kg/m^2^; *p* = 0.005; females: 0.3 kg/m^2^; *p* = 0.012), fat% (males: 1.5%; *p* = 0.002; females: 0.8%; *p* = 0.037) and FM (males: 1.4 kg; *p* = 0.001; females: 0.8 kg; *p* = 0.026). For WC a significant increase was found in males (1.6 cm; *p*<0.001), while females showed a trend towards a significant increase (0.6 cm; *p* = 0.055) between year 4 and 5 (T4-T5). No changes were observed for FFM (males: *p* = 0.719; females: *p* = 0.572) during this final measuring period.

## Discussion

The purpose of this study was to expand on previous research [4] and to provide follow-up results on weight and fat gain among Belgian university students up to five years at university. Male students gained more weight (5.7 kg) than their female counterparts (2.2 kg) over the course of a full university trajectory. These weight gains correspond to increases in BMI of 1.6 and 0.8 kg/m^2^, respectively. In comparison, a US [12] and Canadian [13] study both investigating weight gain from freshman through senior year of college (= 4 years), respectively reported weight (and BMI) gains of 4.2 kg (1.1 kg/m^2^) and 4.1 kg (1.1 kg/m^2^) among males and 1.7 kg (0.5 kg/m^2^) and 3.2 kg (1.1 kg/m^2^) among females. The latter two studies, however, did not include body composition measures. Another US study by Gropper and colleagues [3] demonstrated even greater 4-year weight gains of 5.9 kg (~BMI increase of 1.8 kg/m^2^) in males and 1.7 kg (~BMI increase of 0.6 kg/m^2^) in females which cohered with increases in fat%. In line with the latter study, the observed weight gain in our Belgian student sample was accompanied by increases in BMI, fat% and WC. FFM on the other hand only increased during the first 2.5 years among male but not female students. This FFM increase may be due to maturation (the effect of normal growth and development) and the increase in FM (which implies extra weight the body has to carry around, possibly causing extra growth in muscle mass [14]). These results indicate that the observed weight gain is due to gains in FM more than FFM, which may lead to increased risks for non-communicable diseases, such as cardiovascular disease, type 2 diabetes, etc. in later life [15–17].

Although the statistical significance of the observed weight gains is obvious, it is at least as important to consider the clinical relevancy of such weight gains. In the review of Stevens and colleagues [11] it was recommended to define weight gains of ≥5% (of the initial body weight) as potentially clinically relevant. More than half (53%) of participants in the present study showed clinically relevant weight gains. This illustrates the clinical significance of our results which once again highlights the potential health risks university students may be facing in the future.

Results of the present study showed higher gains in fat% for females (2.5%) compared to males (1.0%), while for WC the inverse was established (males: 4.7 cm vs. females: 1.0 cm). This discrepancy can be explained by the fact that females tend to accumulate fat mostly in the gluteofemoral area (i.e. around the hip), while males usually accumulate fat in the abdominal area (i.e. around the waist) [18]. In the present study hip circumference was not measured, as especially abdominal fat (measured through WC) is strongly related to cardiovascular disease risk in later life [19].

For male students, the present results illustrate a steep increase for weight, BMI, fat%, FM and WC during the first semester, while stabilising (weight, BMI and WC) or returning more or less to baseline (fat% and FM) afterwards. Interestingly, the slope gets steeper again when approaching graduation. For example, this translates into weight increases of 325g/month during the first semester, 117g/month between year 1 and 2, 75g/month between year 2 and 3, 58.3g/month between year 3 and 4, and 116.7g/month during the final measuring period. Although less pronounced, a more or less comparable trend can be observed for female students. For example, weight changes are as great as +150g/month during the first semester, -16.7g/month between year 1 and 2, +25g/month between year 2 and 3, +58.3g/month between year 3 and 4, and +66.7g/month during year 4 and 5. Therefore, it can be suggested that interventions should not only focus on first year but also on final year students.

Students transitioning from high school to college or university have to adapt to completely new environments while entering this new life phase [20–22]. This transition is associated with decreases in fruit and vegetable intake, increases in alcohol consumption and decreases in physical activity, causing weight and fat gain among Belgian college and university students [8]. In two reviews, decreasing physical activity was considered to be one of the most important contributors to student weight gain [23, 24]. It is not clear however, why this second steep increase in weight and fat was observed at the end of students’ university careers. One hypothesis may be that, in their last year at university, students have to finish their Master’s thesis, which implies lots of work and reorganisation of their time, possibly causing an instable momentum with regard to their energy balance related behaviours. Second, the more students approach graduation, the more they become independent and self-responsible. Previous qualitative research showed that Belgian university students’ energy balance related behaviour may not always benefit from increased independency and students often prioritize other social activities over e.g. making time to cook healthy or participating in sports activities [21, 22]. In contrast with e.g. the US or Australia where sports is an important part of (higher) education, in Belgium, students are mainly focused on their study and to a lesser extent on sports activities. Universities should take responsibility by providing healthy environments and encouraging students to make healthy choices both on and off campus. Third, given the fact that young adults stop growing around the age of 21 [25], it may be that they do not immediately adapt their energy balance related behaviours to their ‘new’ energy needs, which may cause an energy imbalance and thus extra increases in weight and fat.

With this notable increase right before graduation the question rises whether or not this weight and fat gain trend will continue after graduation. More importantly, the transition from college or university to work life may also be described as a critical life phase in which people again have to adapt to new environments. This observation opens a new entry point for future research as, to the best of our knowledge, studies investigating the transition from college or university to work life are lacking.

A first strength of the current study is that this is the first European study assessing changes in objectively measured weight and body composition from freshman through senior year of university. Combining both weight and body composition measures allowed us to determine whether gains in weight were attributable to gains in FM or FFM. The measurement of WC allowed us to study changes in body fat distribution. Secondly, given the unique longitudinal data set (including six measuring points over a full academic student trajectory) multilevel modelling was used for data analysis. Multilevel modelling is particularly well fitted to analyse repeated measures over time in a hierarchical structure (i.e. students clustered within study disciplines) including missing values [10]. By including both the individual and study discipline level, the model controls for possible clustering effects [10]. A first limitation of this study is the fact that all participating students were volunteers, which may have resulted in a selection bias. It might be that more healthy students participated in this study. However, sample anthropometrics showed sufficient variance, while representativeness was demonstrated for sex and BMI. Second, we did not account for living arrangements, while students living away from home may be at particular risk for weight and fat gain [7, 26, 27]. Although we asked students about their living arrangements upon study enrolment, we do not have that information across the entire measuring period. As students’ living arrangements may shift throughout university, the inclusion of baseline living arrangements as a confounding variable would deliver biased results. A third limitation of this study is the lack of assessment of energy balance related behaviours. We were therefore not able to relate our findings to changes in e.g. dietary intake, physical activity or sedentary behaviour. Such measures could have uncovered specific causes of these weight and fat gains throughout university.

## Conclusions

Belgian university students gain a significant amount of body weight and body fat from freshman through senior year of university. Male students gain more weight (5.7 kg; corresponding BMI increase = 1.6 kg/m^2^) than females (2.2 kg; corresponding BMI increase = 0.8 kg/m^2^) while increases in fat% were higher in females (2.5%) than males (1%). Further, similar to the first semester more pronounced increases in weight and fat were observed during the final year of university. Interventions aiming at preventing weight gain at university should not only focus on freshmen but also on senior students.

## Supporting information

S1 File Dataset. (XLSX)
